# Radiofrequency ablation versus surgical resection in colorectal liver metastasis: insight from an umbrella review

**DOI:** 10.3389/fonc.2025.1494996

**Published:** 2025-04-04

**Authors:** Letizia Todeschini, Miriam Caimano, Amelia Mattia, Luca Cristin, Alessandro Martinino, Giuseppe Bianco, Gabriele Spoletini, Francesco Giovinazzo

**Affiliations:** ^1^ Faculty of Medicine and Surgery, University of Verona, Verona, Italy; ^2^ Department of Surgery, Fondazione Policlinico Universitario Agostino Gemelli IRCCS, Rome, Italy; ^3^ Department of Surgery, Duke University, Durham, NC, United States; ^4^ UniCamillus-Saint Camillus International University of Health Sciences, Rome, Italy; ^5^ Department of Surgery Saint Camillus Hospital, Treviso, Italy

**Keywords:** colorectal liver metastasis, radiofrequency ablation, surgical resection, survival outcomes, umbrella review

## Abstract

**Introduction:**

Radiofrequency ablation (RFA) has emerged as a less invasive alternative to surgical liver resection (LR) for the treatment of colorectal liver metastasis (CRLM) in patients who are not candidates for surgery. This umbrella review aimed to compare the effectiveness of RFA and LR in managing CRLM by synthesizing evidence from multiple meta-analyses.

**Methods:**

We conducted a comprehensive search across Medline, Epistemonikos, Scopus, and the Cochrane Library, focusing on survival outcomes, disease-free survival, perioperative complications, and recurrence rates.

**Results:**

Eleven meta-analyses met the inclusion criteria. The results show that LR is superior to RFA in terms of overall survival and disease-free survival for resectable CRLM, although RFA demonstrated lower perioperative complications and mortality. In matched cohorts, the overall survival rates between RFA and LR were comparable. However, RFA was associated with higher intrahepatic recurrence.

**Discussion:**

This review highlights the continued importance of LR for resectable CRLM, while RFA remains a valuable option for non-resectable cases, particularly in patients with higher morbidity. Future studies should focus on more balanced cohort comparisons to better assess the efficacy of these treatments.

**Systematic Review Registration:**

https://www.crd.york.ac.uk/PROSPERO/view/CRD42024497886, identifier (CRD42024497886).

## 1 Introduction

Colorectal cancer (CRC), the third most common cancer globally, presents a growing challenge with its increasing disease burden (1). Approximately 20-30% of CRC patients are diagnosed with synchronous liver metastases (CRLM), the most prevalent form of liver malignancy (2). Moreover, around 40% will experience the onset of metachronous liver metastases as the disease advances (3). Surgical liver resection (LR) remains the gold standard in treating CRLM (4). However, its applicability is often limited by operational challenges, patient performance status, and comorbid conditions (4, 5). In such contexts, radiofrequency ablation (RFA) has emerged as a promising technique in the treatment of patients with colorectal liver metastasis (6). RFA presents as an efficacious alternative, offering targeted ablation of metastatic lesions with the advantages of a minimally invasive approach and reduced complication rates (7). Its effectiveness is predominantly observed in cases with limited metastatic burden, optimally in patients harboring a solitary metastasis or multiple metastases, each confined to a size of no more than 3 cm (8). Nonetheless, integrating RFA with surgical resection is recommended to achieve a complete tumor clearance (R0 resection), or as a liver-sparing strategy in scenarios where surgical resection is challenged by difficult tumor locations (9). For instance, this combined approach can improve local control of the tumor in patients with multiple liver lesions (≥4), or in cases where tumors are located near major blood vessels (10). In the latter case, the ‘heat sink effect’ - where flowing blood absorbs and dissipates heat, preventing the tissue from reaching the necessary temperature for complete tumor destruction - can reduce the efficacy of RFA alone, making its combination with surgical resection a more effective strategy (11). However, the use of RFA can be constrained when treating excessively large lesions or those near large blood vessels (9). Additionally, the long-term outcomes of RFA compared to surgery are yet to be established, as evidenced by the lack of published randomized controlled trials. These limitations may have restricted the broader clinical application of RFA.

The aim of the present umbrella review was to systematically evaluate and synthesize the existing evidence from multiple systematic reviews and meta-analyses comparing the role of RFA to surgical resection in the treatment of CRLM.

## 2 Materials and methods

We performed an umbrella review, a comprehensive and systematic evaluation of multiple systematic reviews and meta-analyses, to assess a broad range of postoperative outcomes by synthesizing data on various surgical treatments in CRLM patients (12). This umbrella review was conducted and reported in accordance with the Preferred Reporting Items for Systematic Reviews and Meta-Analyses (PRISMA) guidance (13). Review protocol was registered with the PROSPERO international prospective register of systematic reviews (CRD42024497886).

### 2.1 Search strategy

A computerized search of Medline, Epistemonikos, and Scopus databases, as well as the Cochrane Library, was conducted. Articles published from the time of inception to December 2024 were included. An advanced search was performed with the following search terms: “colorectal neoplasms”, “liver neoplasms”, “liver metastasis”, “treatment”, “systematic review”, “meta-analysis” (**Supplementary Data 1**). Reference lists of all obtained and relevant articles were manually screened and cross-referenced to identify any additional studies by two independent authors (L.T., M.C.). Only articles that evaluated RFA as a treatment option were selected. All published meta-analyses in the English language were included.

### 2.2 Study selection

The search results were imported into the research collaboration software Rayyan (14). L.T, A.M (Amelia Mattia) and L.C. independently screened the meta-analysis by reviewing titles and abstracts. Only articles that compared RFA + LR with hepatic resection in subjects with a diagnosis of CRLM who were older than 18 years were included. Furthermore, included articles had to report survival analysis results in terms of hazard ratio (HR), odds ratio (OR), or relative risk (RR). Only articles in the English language were screened. Conflicts were resolved through discussion with a fourth author A.M. (Alessandro Martinino) and a complete agreement was reached. Full text versions of the articles included after title/abstract screening were obtained and reviewed by the same reviewers.

### 2.3 Data extraction and statistical analysis

Data encompassing authors, publication year, study types, and the number of studies analyzed were extracted by L.T., A.M. (Amelia Mattia) and L.C. for data analysis. Data pertaining to the cohorts of patients involved, including study arms and the number of patients for each of them, was also collected. Additionally, a thorough characterization of the disease state within the included patient cohorts was undertaken to ensure a more refined stratification of the results. Data concerning survival outcomes associated with the chosen treatment were collected as it represents the primary endpoint of our study. Furthermore, other secondary endpoints were extracted to comprehensively assess the benefits of this technique, including disease-free survival (DFS), perioperative mortality, postoperative complications, and rates of intrahepatic recurrence. Pooled outcome measures with 95% confidence interval values (95%CI), statistical heterogeneity and publication bias were also calculated. A.M. (Alessandro Martinino) examined all extractions conducted by the three authors, ensuring that the extraction criteria were consistently applied according to the predetermined standards set before the extraction commenced.

### 2.4 Data analysis

Qualitative summary of the results, using text and tables, was performed. To ensure consistency, standardization and comparability across studies we re-extracted effect sizes, and recalculated pooled effect sizes together with heterogeneity measures (I², τ²) (12). We combined trials from previously published meta-analyses into updated meta-analyses after removing duplicated trials. Effect size for dichotomous outcomes was expressed as Risk Ratio (RR) and Odd Ratio (OR) with their 95% of confidence interval (CI). Hazard Ratio (HR) with standard error (SE) were used for time-to-event outcomes. A p-value < 0.05 (two-tailed) was considered to indicate statistical significance. We planned the meta-analysis if there were 2 or more studies with the same outcome. Heterogeneity was assessed using the I^2^ statistic. I^2^ values greater than 50% suggesting significant heterogeneity, therefore a random-effect meta-analysis was performed otherwise a fixed-effect model. All statistical analyses were done with RevMan 5, version 5.4.

### 2.5 Quality assessment

The internal validity of the meta-analyses was assessed by the Assessment of Multiple Systematic Reviews 2 (AMSTAR-2) method. L.T. and M.C. completed the 11-items AMSTAR proforma for all included meta-analyses, and discrepancies were discussed to reach a consensus with a third author (A.M.) (15). Finally, studies were classified based on the level of quality through the online tool calculator.

## 3 Results

### 3.1 Study selection

**Figure 1:**
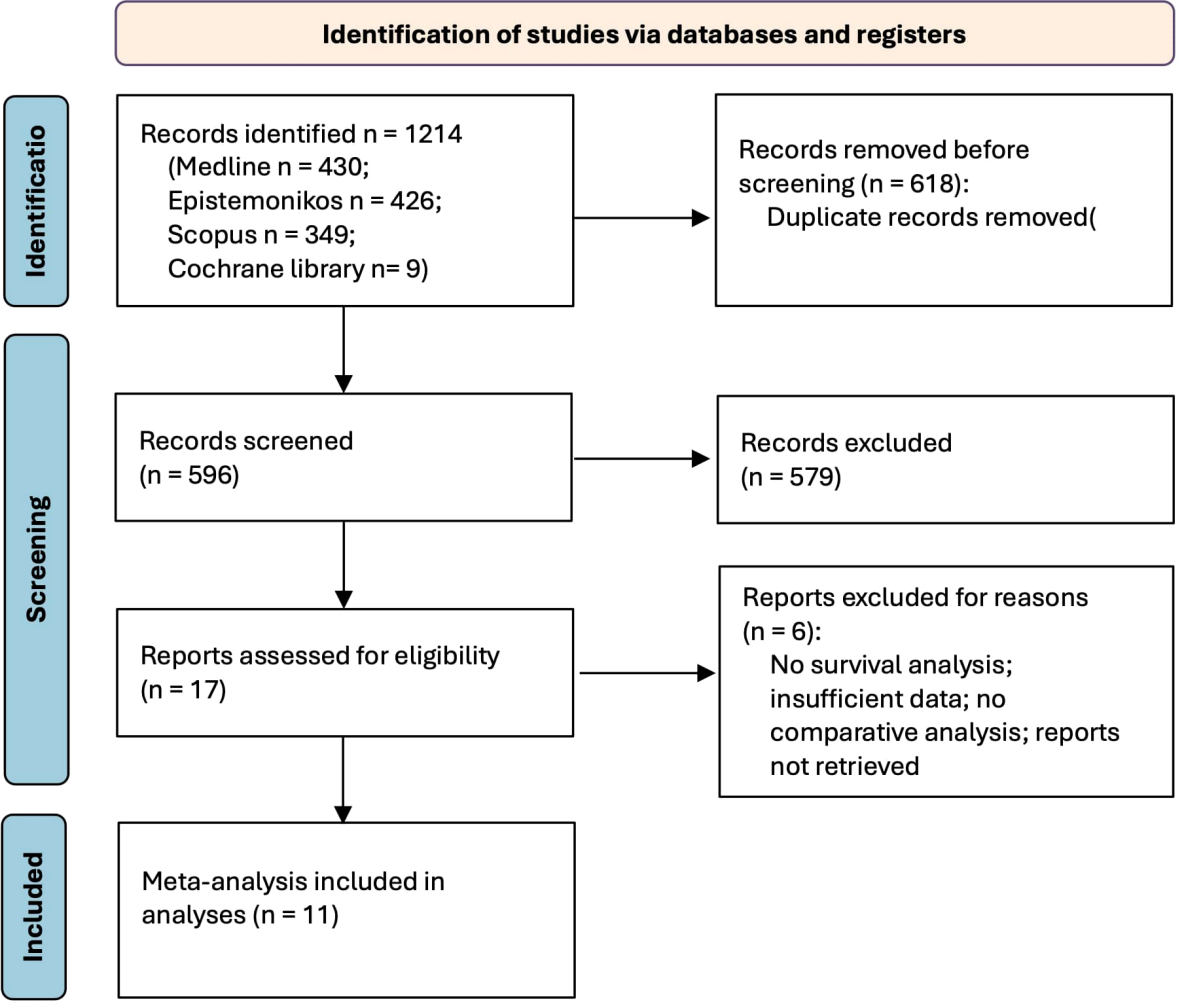
PRISMA flow diagram.

A total of 1214 potentially relevant articles were identified using the search strategy described in the Method section. 618 duplicate records were removed, leaving 596 records available for screening. A total of 579 articles were excluded after abstract reviewing due to inappropriate topic relevance, absence of a meta-analysis, and being in a language other than English. Among the remaining 17 articles, 3 were excluded due to full text not available, and another 3 were excluded by examining the full texts because of the absence of survival analysis, comparative analysis, or insufficient data. Finally, 11 meta-analyses met the inclusion criteria and were included in the present umbrella review (16–26). A summary of the results of the systematic search is shown in the PRISMA 2020 flow diagram (**Figure 1**).

### 3.2 Quality assessment results

Quality levels of the meta-analyses included are reported in the last column of **Table 1**. Among the 11 meta-analyses included in our umbrella review, all of them demonstrated a critically low quality according to the AMSTAR-2 evaluation. The main common reasons for such low quality were the absence of a registered protocol, the lack of an appropriate risk of bias (RoB) assessment, and a strong heterogenicity among samples, with differences in the number and size of ablated lesions, the approaches to ablation (which range from percutaneous to open or laparoscopic), and the specifics of the ablative therapies.

**Table 1:** The bold text was used to highlight the authors of the papers considered for easier identification. However, it can absolutely be removed to avoid confusion.

Author, Year	Primary studies design (n)	Purpose	N° of participants (n)	Condition	Comparison	Outcome (n studies)	Metrics [95%CI]	P-value	I^2^ (%)	Quality
Wu YZ, 2011 (16)	R (7)	To evaluate the comparative therapeutic efficacy of RFA and LR for solitary colorectal liver metastases	847	Solitary CRLM	RFA vs LR	5-y OS (7)	OR: 0.41 [0.22, 0.90]	0.008	64.2	Critically low
Weng M, 2012 (17)	R (12) P (1)	To summarize the evidence mostly from clinical trials and to investigate the effect of LR and RFA	1886	CRLM	LR vs RFA	5-y OS (11)	RR: 1.474 [1.284, 1.692]	<0.001	21.7	Critically low
						5-y DFS (10)	RR: 2.227 [1.823, 2.720]	<0.001	71.8	
Bai H, 2015 (18)	R (9) P (1)	To pool available evidence and to analyze the effect of LR and RFA for resectable solitary CRLM in survival indicators	1911	CRLM	LR vs RFA	OS (10)	HR: 1.85 [1.48, 2.32]	<0.001	47	Critically low
						DFS (8)	HR: 1.68 [1.14, 2.48]	0.009	78	
Han Y, 2016 (19)	R (14)	To summarize the related clinical evidences about the therapeutic value of RFA and LR in the treatment of colorectal cancer liver metastases	2205	CRLM	LR vs RFA	5-y OS (13)	HR: 1.361 [1.163, 1.593]	<0.001	73.2	Critically low
						5-y PFS (11)	HR: 1.396 [1.230, 1.584]	<0.001	81.2	
Van Amerongen MJ, 2017 (20)	R (20)	To systematically evaluate the role of RFA compared to surgery in the curative treatment of patients with colorectal liver metastases	2877	CRLM	RFA vs LR	5-y OS (13)	OR: 2.35 [1.49, 3.69]	<0.001	74	Critically low
						5-y DFS (9)	OR: 2.20 [1.28, 3.79]	0.005	62	
Long L, 2018 (21)	R (10)	To compare long-term outcomes after combined LR and RFA with conventional LR for CRLM	3900	CRLM	LR vs RFA + LR	OS (10)	HR: 2.07 [1.82, 2.37]	<0.001	64.7	Critically low
						DFS (7)	HR: 1.91 [1.70, 2.15]	<0.001	68.1	
Meijerink MR, 2018 (22)	R (21)	To assess safety and outcome of RFA and MWA as compared to systemic chemotherapy for unresectable disease and LR for resectable disease	5020	Resectable and unresectable CRLM	RFA vs LR	OS (10)	HR: 1.78 [1.35, 2.33]	<0.001	59	Critically low
						LPFS (3)	HR: 5.36 [1.64, 17.52]	0.005	75	
						DFS (5)	HR: 1.49 [1.23, 1.81]	<0.001	10	
					RFA + HR vs LR	OS (7)	HR: 1.24 [0.84, 1.84]	0.28	77	
						LPFS (2)	HR: 1.64 [1.22, 2.20]	<0.001	0	
						DFS (4)	HR: 1.14 [0.82, 1.60]	0.44	63	
Di Martino M, 2020 (23)	R (6) P (3)	To evaluate local ablative therapies and compare them with surgical resection	244	CRLM	RFA vs LR	5y DFS (5)	RR: 0.43[0.21, 0.88]	0.02	27	Critically low
						5y OS (4)	RR: 0.69 [0.51, 0.93]	0.02	0	
Hao W, 2020 (24)	R (10)	To evaluate noninferiority or inferiority of RFA compared to LR for patients with unresectable CRLM	1037	Solitary CRLM	RFA vs. LR	1-y PFS (10)	RR: 0.77 [0.630, 0.940]	0.009	86	Critically low
						5-y OS (10)	RR: 0.66 [0.52, 0.85]	0.001	55.7	
Yang G, 2021 (25)	R (22)	To compare the prognosis of RFA and LR in treatment of CRLM	4385	CRLM	RFA vs LR	5-yr OS (15)	OR: 0.60 [0.48, 0.74]	<0.001	17	Critically low
						5-yr DFS(8)	OR: 0.74 [0.56, 0.97]	0.03	35	
Gavriilidis P, 2021 (26)	R (26)	To compare the local recurrence rate and long-term survival in patients undergoing LR, RFA or MWA	3401	CRLM	RFA vs LR	5-year DFS (10)	HR: 1.30 [1.09, 1.55]	0.003	72	Critically low
						5-year OS (15)	HR: 1.40 [1.21, 1.64]	< 0.001	52	

RFA, radiofrequency ablation; LR, liver resection; MWA, microwave ablation; CLRM, colorectal liver metastasis; CI, confidence interval; OS, overall survival; DFS, disease-free survival; HR, hazard ratio; OR, odds ratio; RR, relative risk.

### 3.3 Study characteristics

A total of 11 meta-analyses were included into our study (**Table 1**). The included patients all had a diagnosis of CRLM, with two meta-analyses specifically concentrating on patients with solitary CRLM (16, 24). Hepatic lesions were < 5 cm on average. Of the 11 meta-analyses, 9 compared RFA with LR exclusively (16–20, 23–26), one evaluated RFA against a combined approach of LR and RFA (21), and one study addressed both comparisons (22). As per inclusion criteria, all the meta-analyses included in our study examined survival outcomes, which were reported as OS, 3-years OS, and 5-years OS. A wide range of secondary outcomes were investigated: DFS, 3-years DFS, 5-years DFS, perioperative mortality, postoperative complications, recurrence, new intrahepatic recurrence, marginal recurrence, distant recurrence.

### 3.4 Survival outcomes

In our umbrella meta-analyses, we evaluated survival outcomes in patients who underwent RFA or LR (**Table 2**). OS comparison between patients receiving RFA and patients undergoing LR revealed a significant survival benefit in favor of LR (HR 1.73; 95% C.I. 1.39-2.16; p < 0.001; I^2^ 57%). Additionally, 3-year OS data also indicated a superior survival rate for those treated with LR compared to RFA (OR 1.68; 95% C.I. 1.11-2.54; p = 0.01; I^2^ 56%). When analyzing 5-year OS, our meta-analysis showed a significant advantage was identified in the LR group compared to the RFA group (OR 2.14; 95% C.I. 1.49-3.06; p < 0.001; I^2^ 68%). Similarly, when considering only those primary studies that evaluated solitary hepatic lesions, better survival was observed in the LR group compared to the RFA group (HR 1.82; 95% C.I. 1.24-2.68; p = 0.002; I^2^ 57%) (**Table 3**). A survival analysis considering only studies who performed a matched cohort analysis was conducted (**Table 4**). This analysis included data from 3 primary studies comparing RFA with LR. The findings indicated a trend towards better survival in patients treated with LR; however, this difference was not statistically significant (HR 1.26; 95% C.I. 0.99-1.62; p = 0.06; I^2^ 0%).

**Table 2:** Meta-analyses results: RFA vs LR.

Parameter	Meta-analyses included (n)	Primary studies included (n)	Patients in the RFA group (n)	Patients in the LR group (n)	Metrics [95% CI] recalculated	P value	I2 (%)
RFA vs LR							
OS	4	14	944	1448	HR: 1.73 [1.39, 2.16]	< 0.001	57
DFS	4	14	949	1458	HR: 1.65 [1.33, 2.05]	< 0.001	67
3-yr OS	6	11	367	1012	OR: 1.68 [1.11, 2.54]	0.01	56
5-yr OS	8	20	1016	1574	OR: 2.14 [1.49, 3.06]	< 0.001	68
3-yr DFS	3	4	126	293	OR: 1.69 [1.08, 2.66]	0.02	16
5-yr DFS	5	14	828	1178	OR: 3.13 [1.93, 5.08]	< 0.001	66
Perioperative mortality	4	14	875	1165	RR: 0.79 [0.39,1.59]	0.51	0
Postoperative complications	5	17	1027	1335	RR: 0.60 [0.42, 0.84]	0.003	63
Recurrence	4						
New intrahepatic recurrence		17	922	1566	RR: 1.89 [1.48, 2.40]	< 0.001	63
Marginal recurrence		20	1034	1800	RR: 4.94 [3.82, 6.39]	< 0.001	5
Distant recurrence		19	1002	1740	RR: 0.93 [0.76, 1.14]	0.49	58
RFA+LR vs LR							
OS	2	8	439	1658	HR: 1.34 [0.95, 1.89]	0.09	71
DFS	2	8	439	1658	HR: 1.26 [1.01, 1.57]	0.04	55
3-yr OS	1	4	265	917	OR: 3.12 [2.34, 4.17]	< 0.001	0
5-yr OS	2	4	288	1363	OR: 1.02 [0.34, 3.06]	0.97	93
5-yr DFS	2	3	202	964	OR: 1.25 [0.45, 3.42]	0.67	86
Postoperative complications	2	4	236	1185	RR: 1.13 [0.89, 1.43]	0.32	47
Recurrence	2						
New intrahepatic recurrence		6	389	1439	RR: 1.66 [1.07, 2.56]	0.02	89
Marginal recurrence		6	389	1439	RR: 2.47 [1.72, 3.56]	< 0.001	0
Distant recurrence		5	309	1159	RR: 1.27 [0.87, 1.86]	0.21	79

RFA, radiofrequency ablation; LR, liver resection; CI, confidence interval; OS, overall survival; DFS, disease-free survival; HR, hazard ratio; OR, odds ratio; RR, relative risk.

**Table 3:** Sub-meta-analysis on patients with a single hepatic lesion.

Parameter	Primary studies included (n)	Metrics [95% CI] recalculated	P value	I^2^ (%)
OS	6	HR: 1.82 [1.24, 2.68]	0.002	57
DFS	5	HR: 1.79 [1.27, 2.54]	0.001	56

RFA, radiofrequency ablation; LR, liver resection; CI, confidence interval; OS, overall survival; DFS, disease-free survival; HR, hazard ratio; OR, odds ratio; RR, relative risk.

**Table 4:** Meta-analysis of matched cohort studies.

Parameter	Primary studies included (n)	Metrics [95% CI] recalculated	P value	I^2^ (%)
**OS**	3	HR: 1.26 [0.99, 1.62]	0.06	0
**DFS**	4	HR: 1.42 [1.15, 1.75]	0.001	0

RFA, radiofrequency ablation; LR, liver resection; CI, confidence interval; OS, overall survival; DFS, disease-free survival; HR, hazard ratio; OR, odds ratio; RR, relative risk.

Similar survival outcomes were obtained comparing a combination of RFA and LR (RFA+LR) with surgery alone (**Table 2**). The comparison of OS in patients receiving RFA+LR versus LR alone, did not reach a statistical relevance (HR 1.34; 95% C.I. 0.95-1.89; p = 0.09; I^2^ 71%). 3-year OS outcomes were reported in one meta-analysis, that demonstrated a higher survival rate for patients in the LR group (OR 3.12; 95% C.I. 2.34-4.17; p < 0.001; I^2^ 0%). Lastly, for 5-year OS data not statistical relevance was observed (OR 1.02; 95% C.I. 0.34-3.06; p = 0.97; I^2^ 93%).

### 3.5 Secondary outcomes

The results of the meta-analysis specifically examining the secondary outcomes from the RFA and LR groups, are comprehensively presented in **Table 2**. DFS analysis yielded a statistically significant advantage for LR compared to RFA (HR 1.65, 95% C.I. 1.33-2.05; p < 0.001; I^2^ 67%). A similar trend was in the comparison of 5-year DFS, where the LR group exhibited more favorable outcomes (OR 3.13; 95% C.I. 1.93-5.08; p < 0.001; I^2^ 66%). Further substantiating these findings, a sub-analysis focusing solely on patients with solitary hepatic lesions revealed an enhanced DFS in the LR group in comparison to the RFA group (HR 1.79; 95% C.I. 1.27-2.54; p = 0.001; I^2^ 56%) (**Table 3**). Moreover, the matched cohort analysis studies corroborated these results, demonstrating a higher DFS in the LR group (HR 1.42; 95% C.I. 1.15-1.75; p = 0.001, I^2^ 0%) (**Table 4**).

When examining perioperative mortality, the RR associated with patients undergoing RFA compared to those undergoing LR was 0.79 (95% C.I. 0.39-1.59); however, this finding did not achieve statistical significance (p = 0.51). The comparison between patients undergoing RFA and LR patients revealed a significant RR of 0.60 (95% CI 0.42-0.84; p = 0.003; I^2^ 63%) for developing postoperative complications. Lastly, exploration of intrahepatic recurrence rate in RFA patients and LR patients, indicated a substantial higher risk in the first group. In fact, we observed a significantly higher risk of new intrahepatic recurrence in patients treated with RFA compared to LR (RR 1.89; 95% C.I. 1.48-2.40; p < 0.001; I^2^ 63%). Similarly, the risk of marginal recurrence was notably higher in the RFA group compared to the LR group (RR of 4.94; 95% CI 3.82-6.39; p < 0.001, I^2^ 5%). However, when it came to distant recurrence, the difference between the two operative modalities was not statistically significant (RR 0.93; 95% CI 0.76-1.14; p = 0.49; I^2^ 58%).

Comparison between RFA+LR and surgery alone yielded similar results for all the parameters above (**Table 3**). DFS was higher in surgery alone group than in the combination therapy group (HR 1.26; 95% CI 1.01-1.57; p = 0.04; I^2^ 55%). For 5-years DFS, statistical relevance was not achieved (OR 1.25; 95% C.I. 0.45-3.42; p = 0.67; I^2^ 86%). Postoperative complications also did not show statistically significant differences between the two groups (RR 1.13; 95% C.I. 0.89-1.43; I^2^ 86%; p = 0.32). As before, we also observed that for new intrahepatic recurrence, the patients receiving the combined RFA and LR treatment exhibited a significantly higher risk compared to the patients undergoing LR alone (RR 1.66; 95% CI 1.07-2.56; p = 0.02; I^2^ 89%). In the case of marginal recurrence, the same cohort of patients displayed an even more pronounced increased risk (RR 2.47; 95% CI 1.72-3.56; p < 0.001; I^2^ 0%). When assessing distant recurrence, the difference between the two operative modalities was not statistically significant (RR 1.27; 95% CI 0.87-1.86; p = 0.21, I^2^ 79%).

## 4 Discussion

Surgical resection is the preferred initial treatment for CRLM, but it may not be feasible in cases of challenging anatomical locations or poor patient health (5). RFA is a minimally invasive alternative that has emerged as a recognized and viable treatment option for small-sized CRC liver metastases (27–29). This technique employs a needle that administers an alternating current to produce high heat for tumor destruction, now enhanced by innovative devices featuring expandable electrodes or internally cooled tips (30–32). Despite its increasing utilization in clinical practice, certain factors can restrict its adoption including large or numerous lesions, or those situated near large blood vessels or the surface of the liver, which can compromise the effectiveness of the procedure and heighten the likelihood of complications (33, 34). Moreover, the long-term efficacy and safety of RFA in the treatment of CRLM compared to surgical resection remain a subject of ongoing debate within the literature. Most of the existing evidence on the efficacy of RFA for CRC liver metastases is derived from retrospective studies. Many of these studies are characterized by limited follow-up periods, often not exceeding 20 months, posing challenges in conclusively determining the long-term outcomes of RFA treatment. The results of our umbrella meta-analyses provide valuable insights into the comparative efficacy of RFA and LR in treating hepatic metastases of CRC, with data encompassing up to a long-term period of 5 years.

A significant survival benefit was observed in patients undergoing LR compared to those who had RFA. This was evident in both short-term (3-year OS) and long-term (5-year OS) follow-ups. The superiority of LR was also pronounced in patients with solitary hepatic lesions. These results suggest that RFA, although being a less invasive technique, might not offer a survival benefit. When comparing a combination of LR and RFA (LR+RFA), a survival advantage was observed in the group undergoing only resection. While this advantage was not always statistically significant, it suggests that RFA may not play a significant role in enhancing survival.

The analysis of DFS demonstrates a clear advantage for LR. This benefit is evident when comparing DFS outcomes of LR against implementation of RFA, whether the latter is used alone or in conjunction with surgery. This is particularly notable in the 5-year DFS comparisons, where LR alone shows a statistically significant benefit. The distinction is less pronounced when comparing LR to a combination treatment with RFA, yet it still indicates a trend towards improved outcomes.

Regarding perioperative mortality, our data did not reveal significant differences between RFA and surgical treatments. However, what stands out is the markedly lower rate of postoperative complications observed in the RFA group. This trend persists even when RFA is used in conjunction with surgery, suggesting a consistently reduced risk of complications. This aspect of our findings is particularly significant, as it suggests that RFA, either alone or in combination with surgery, might offer a safer treatment path with potentially less intensive postoperative care requirements. This lower complication profile could be a decisive factor in treatment choice, especially in patients where minimization of postoperative risk is a primary concern. However, a critical finding was the substantially higher rate of intrahepatic recurrence in patients treated with RFA. This suggests that while RFA may offer better initial outcomes in terms of perioperative complications, there is a higher risk of recurrence, which could impact long-term survival and quality of life. As RFA resembles the treatment effect of a wedge (i.e., non-anatomical) liver resection, this result confirms the previous evidence that the anatomical removal of the portal tributaries of a given metastasis reduces the risk of liver recurrence after surgery (35, 36). Notably, the risk of recurrence may be influenced by several prognostic factors, including bilobar disease, positive surgical margins, and elevated tumor markers (37). Additionally, molecular markers such as RAS, BRAF, and SMAD4 mutations significantly impact overall and recurrence-free survival, particularly in patients undergoing liver metastasectomy (38). These genetic factors may explain the higher intrahepatic recurrence rates observed after RFA, reinforcing the importance of careful patient selection. At the same time, technical factors remain crucial. Studies have shown that achieving an ablative margin greater than 5 mm during RFA significantly reduces local tumor progression (39). Advanced imaging techniques, particularly contrast-enhanced MRI, can improve treatment planning by providing a more accurate assessment of tumor extent and guiding surgical or ablative approaches (40). Ensuring precise margins, whether through surgery or locoregional therapies, is key to minimizing recurrence risk. Ultimately, optimizing CRLM treatment requires a multidisciplinary approach. The decision between surgery, RFA, or other locoregional therapies should balance both technical feasibility and tumor biology. While advancements in imaging and ablation techniques have improved outcomes, their success still depends on careful planning and patient selection.

A critical limitation of our study, and indeed a potential confounding factor in our results, stems from the selection criteria for the patient cohort undergoing RFA. Specifically, these patients were those considered ineligible for surgical resection, and that inherently places them at a survival disadvantage relative to candidates for LR. This aspect suggests that the observed differences in treatment efficacy might not solely reflect the intrinsic merits of the treatment modalities but could also be influenced by the baseline survival prospects of the patient groups involved. Other limitations must be acknowledged as they potentially impact the interpretation of our umbrella review results. The quality of the included meta-analyses, assessed using AMSTAR-2 criteria, was found to be generally critically low, necessitating prudence in the interpretation of their findings. Future studies should aim to mitigate the impact of selection bias arising from patient and tumor characteristics in surgical treatment decisions through rigorous sensitivity analyses while ensuring methodological robustness by adhering to AMSTAR-2 criteria, thereby strengthening the validity and reliability of evidence in surgical oncology research. Additionally, the studies predominantly comprise non-randomized controlled trial data, including retrospective cohort studies, which could potentially introduce bias. To mitigate these issues, we performed a survival analysis using only matched cohort analysis studies, pairing patients who underwent RFA alone with those who underwent LR alone, based on prognostic characteristics. This approach was designed to simulate the randomization process typical of clinical trials, aiming to minimize selection bias and thereby enhance the reliability of our conclusions. While the analysis of disease-free survival revealed a benefit for surgical resection over RFA, both treatment modalities yielded comparable outcomes in terms of overall survival.

## 5 Conclusions

Our study represents the first umbrella review to compare RFA and LR in the management of CRLM, offering a clear overview of the existing evidence on this topic. As suggested by our results, LR maintains its crucial role in the management of CRLM, given its advantages in both survival and disease-free survival. However, given its significantly lower rate of postoperative complications, RFA stands out as a less invasive treatment approach in CRLM management in selected comorbid patients. Nevertheless, to fully ascertain its impact on survival and quality of life, further long-term follow-up studies and randomized controlled trials are needed. Future investigations should: (1) focus on specific patient subgroups, such as those with solitary CRLM ≤3 cm, to clarify the efficacy of RFA in these scenarios; (2) compare patients with similar baseline characteristics in terms of prognosis to minimize selection bias; (3) employ long-term follow-up studies (beyond 5 years) while investigating causes of higher recurrence rates with RFA. Future systematic reviews and meta-analyses should include more high-quality randomized controlled trials, such as the ongoing COLLISION trial, to compare RFA and LR. Such research would provide a clearer and more definitive comparison of the efficacy of LR versus RFA, thereby offering more reliable guidance for clinical decision-making.

## Data Availability

The original contributions presented in the study are included in the article/**Supplementary Material**. Further inquiries can be directed to the corresponding author.

## References

[1] Adam R De Gramont A Figueras J Guthrie A Kokudo N Kunstlinger F . The oncosurgery approach to managing liver metastases from colorectal cancer: a multidisciplinary international consensus. Oncologist. (2012) 17:1225–39. doi: 10.1634/theoncologist.2012-0121 PMC3481888 22962059

[2] Manfredi S Lepage C Hatem C Coatmeur O Faivre J Bouvier AM . Epidemiology and management of liver metastases from colorectal cancer. Ann Surg. (2006) 244:254–9. doi: 10.1097/01.sla.0000217629.94941.cf PMC1602156 16858188

[3] Arnold M Sierra MS Laversanne M Soerjomataram I Jemal A Bray F . Global patterns and trends in colorectal cancer incidence and mortality. Gut. (2017) 66(4):683–91. doi: 10.1136/gutjnl-2015-310912 26818619

[4] Nieuwenhuizen S Puijk RS van den Bemd B Aldrighetti L Arntz M van den Boezem PB . Resectability and ablatability criteria for the treatment of liver only colorectal metastases: multidisciplinary consensus document from the COLLISION trial group. Cancers (Basel). (2020) 12:1779. doi: 10.3390/cancers12071779 32635230 PMC7407587

[5] Scheele J Stang R Altendorf-Hofmann A Paul M . Resection of colorectal liver metastases. World J Surg. (1995) 19:59–71. doi: 10.1007/BF00316981 7740812

[6] Gillams AR Lees WR . Radiofrequency ablation of colorectal liver metastases. Abdom Imaging. (2005) 30:419–26. doi: 10.1007/s00261-004-0256-6 15759208

[7] Hompes D Prevoo W Ruers T . Radiofrequency ablation as a treatment tool for liver metastases of colorectal origin. Cancer Imaging. (2011) 11:23–30. doi: 10.1102/1470-7330.2011.0004 21435988 PMC3080126

[8] Abitabile P Hartl U Lange J Maurer CA . Radiofrequency ablation permits an effective treatment for colorectal liver metastasis. Eur J Surg Oncol. (2007) 33:67–71. doi: 10.1016/j.ejso.2006.10.040 17174059

[9] Van Cutsem E Cervantes A Adam R Sobrero A Van Krieken JH Aderka D . ESMO consensus guidelines for the management of patients with metastatic colorectal cancer. Ann Oncol. (2016) 27:1386–422. doi: 10.1093/annonc/mdw235 27380959

[10] Masuda T Margonis GA Andreatos N Wang J Warner S Mirza MB . Combined hepatic resection and radio-frequency ablation for patients with colorectal cancer liver metastasis: A viable option for patients with a large number of tumors. Anticancer Res. (2018) 38:6353–60. doi: 10.21873/anticanres.12993 30396957

[11] Huang HW . Influence of blood vessel on the thermal lesion formation during radiofrequency ablation for liver tumors. Med Phys. (2013) 40:073303. doi: 10.1118/1.4811135 23822457

[12] Ioannidis JPA . Integration of evidence from multiple meta-analyses: a primer on umbrella reviews, treatment networks and multiple treatments meta-analyses. CMAJ. (2009) 181:488–93. doi: 10.1503/cmaj.081086 PMC2761440 19654195

[13] Page MJ McKenzie JE Bossuyt PM Boutron I Hoffmann TC Mulrow CD . The PRISMA 2020 statement: an updated guideline for reporting systematic reviews. BMJ. (2021) 372:n71. doi: 10.1136/bmj.n71 33782057 PMC8005924

[14] Ouzzani M Hammady H Fedorowicz Z Elmagarmid A . Rayyan—a web and mobile app for systematic reviews. Systematic Rev. (2016) 5:210. doi: 10.1186/s13643-016-0384-4 PMC5139140 27919275

[15] Shea BJ Reeves BC Wells G Thuku M Hamel C Moran J . AMSTAR 2: a critical appraisal tool for systematic reviews that include randomised or non-randomised studies of healthcare interventions, or both. BMJ. (2017) 358:j4008. doi: 10.1136/bmj.j4008 28935701 PMC5833365

[16] Wu YZ Li B Wang T Wang SJ Zhou YM . Radiofrequency ablation vs hepatic resection for solitary colorectal liver metastasis: A meta-analysis. World J Gastroenterology. (2011) 17:4143–8. doi: 10.3748/wjg.v17.i36.4143 PMC3203368 22039331

[17] Weng M Zhang Y Zhou D Yang Y Tang Z Zhao M . Radiofrequency ablation versus resection for colorectal cancer liver metastases: A meta-analysis. PloS One. (2012) 7:e45493. doi: 10.1371/journal.pone.0045493 23029051 PMC3448670

[18] Bai H Huangz X Jing L Zeng Q Han L . The effect of radiofrequency ablation vs. liver resection on survival outcome of colorectal liver metastases (CRLM): a meta-analysis. Hepatogastroenterology. (2015) 62(138):373–7. 25916066

[19] Han C Liu T Yin R . Biomarkers for cancer-associated fibroblasts. biomark Res. (2020) 8:64. doi: 10.1186/s40364-020-00245-w 33292666 PMC7661188

[20] van Amerongen MJ Jenniskens SFM van den Boezem PB Fütterer JJ de Wilt JHW . Radiofrequency ablation compared to surgical resection for curative treatment of patients with colorectal liver metastases – a meta-analysis. HPB. (2017) 19(9):749–56. doi: 10.1016/j.hpb.2017.05.011 28687147

[21] Long L Wei L Hong W . Meta-analysis of long-term outcomes in patients with colorectal liver metastases undergoing hepatectomy with or without radiofrequency ablation. Am SurgeonTM. (2018) 84:1913–23. doi: 10.1177/000313481808401237 30606348

[22] Meijerink MR Puijk RS Van Tilborg AAJM Henningsen KH Fernandez LG Neyt M . Radiofrequency and microwave ablation compared to systemic chemotherapy and to partial hepatectomy in the treatment of colorectal liver metastases: A systematic review and meta-analysis. Cardiovasc Intervent Radiol. (2018) 41:1189–204. doi: 10.1007/s00270-018-1959-3 PMC6021475 29666906

[23] Di Martino M Rompianesi G Mora-Guzmán I Martín-Pérez E Montalti R Troisi RI . Systematic review and meta-analysis of local ablative therapies for resectable colorectal liver metastases. Eur J Surg Oncol. (2020) 46:772–81. doi: 10.1016/j.ejso.2019.12.003 31862133

[24] Hao W Binbin J Wei Y Kun Y . Can radiofrequency ablation replace liver resection for solitary colorectal liver metastasis? A systemic review and meta-analysis. Front Oncol. (2020) 10:561669. doi: 10.3389/fonc.2020.561669 33312946 PMC7706822

[25] Yang G Wang G Sun J Xiong Y Li W Tang T . The prognosis of radiofrequency ablation versus hepatic resection for patients with colorectal liver metastases: A systematic review and meta-analysis based on 22 studies. Int J Surgery. (2021) 87:105896. doi: 10.1016/j.ijsu.2021.105896 33588125

[26] Gavriilidis P Roberts KJ de’Angelis N Aldrighetti L Sutcliffe RP . Recurrence and survival following microwave, radiofrequency ablation, and hepatic resection of colorectal liver metastases: A systematic review and network meta-analysis. Hepatobiliary Pancreatic Dis Int. (2021) 20:307–14. doi: 10.1016/j.hbpd.2021.05.004 34127382

[27] Gillams AR . Thermal ablation of liver metastases. Abdom Imaging. (2001) 26:361–8. doi: 10.1007/s002610000196 11441547

[28] Gillams AR Lees WR . Survival after percutaneous, image-guided, thermal ablation of hepatic metastases from colorectal cancer. Dis Colon Rectum. (2000) 43:656–61. doi: 10.1007/BF02235582 10826427

[29] Montgomery RS Rahal A Dodd GD Leyendecker JR Hubbard LG . Radiofrequency ablation of hepatic tumors: variability of lesion size using a single ablation device. AJR Am J Roentgenol. (2004) 182:657–61. doi: 10.2214/ajr.182.3.1820657 14975966

[30] Cirocchi R Trastulli S Boselli C Montedori A Cavaliere D Parisi A . Radiofrequency ablation in the treatment of liver metastases from colorectal cancer. Cochrane Database Syst Rev. (2012) (6):CD006317. doi: 10.1002/14651858.CD006317.pub3 22696357 PMC11931680

[31] Decadt B Siriwardena AK . Radiofrequency ablation of liver tumours: systematic review. Lancet Oncol. (2004) 5:550–60. doi: 10.1016/S1470-2045(04)01567-0 15337485

[32] McGahan JP Gu WZ Brock JM Tesluk H Darryl Jones C . Hepatic ablation using bipolar radiofrequency electrocautery. Acad Radiology. (1996) 3:418–22. doi: 10.1016/S1076-6332(05)80677-4 8796695

[33] Lu DSK Raman SS Limanond P Aziz D Economou J Busuttil R . Influence of large peritumoral vessels on outcome of radiofrequency ablation of liver tumors. J Vasc Interv Radiol. (2003) 14:1267–74. doi: 10.1097/01.RVI.0000092666.72261.6B 14551273

[34] Machi J Uchida S Sumida K Limm WM Hundahl SA Oishi AJ . Ultrasound-guided radiofrequency thermal ablation of liver tumors: percutaneous, laparoscopic, and open surgical approaches. J Gastrointest Surg. (2001) 5:477–89. doi: 10.1016/S1091-255X(01)80085-8 11985998

[35] Margonis GA Buettner S Andreatos N Sasaki K Ijzermans JNM van Vugt JLA . Anatomical resections improve disease-free survival in patients with KRAS-mutated colorectal liver metastases. Ann Surg. (2017) 266:641–9. doi: 10.1097/SLA.0000000000002367 28657938

[36] Chang W Chen Y Zhou S Ren L Xu Y Zhu D . Anatomical resection improves relapse-free survival in colorectal liver metastases in patients with KRAS/NRAS/BRAF mutations or right-sided colon cancer: a retrospective cohort study. Int J Surg. (2023) 109:3070–7. doi: 10.1097/JS9.0000000000000562 PMC10583959 37526097

[37] Tian Y Wang Y Wen N Wang S Li B Liu G . Prognostic factors associated with early recurrence following liver resection for colorectal liver metastases: a systematic review and meta-analysis. BMC Cancer. (2024) 24:426. doi: 10.1186/s12885-024-12162-4 38584263 PMC11000331

[38] Roncato R Polesel J Tosi F Peruzzi E Brugugnoli E Pantano CL . The challenge of molecular selection in liver-limited metastatic colorectal cancer for surgical resection: a systematic review and meta-analysis in the context of current and future approaches. Oncol Res. (2024) 32:1407–22. doi: 10.32604/or.2024.049181 PMC11361904 39220128

[39] Verdonschot KHM Arts S Van den Boezem PB de Wilt JHW Fütterer JJ Stommel MWJ . Ablative margins in percutaneous thermal ablation of hepatic tumors: a systematic review. Expert Rev Anticancer Ther. (2023) 23:977–93. doi: 10.1080/14737140.2023.2247564 37702571

[40] Karaoğlan BB Öz DK Araz MS Akyol C Utkan G . Advancements in the management of synchronous colorectal liver metastases: A comprehensive review of surgical, systemic, and local treatment modalities. Curr Oncol Rep. (2024) 26:791–803. doi: 10.1007/s11912-024-01548-z 38776011 PMC11224077

