# Supplementary material for: Radiofrequency ablation versus surgical resection in colorectal liver metastasis: insight from an umbrella review
Source: Front Oncol. 2025 Apr 4;15:1494996. doi: 10.3389/fonc.2025.1494996 (PMC12006127; doi:10.3389/fonc.2025.1494996)
Supplement: Supplementary file 1 [file Table1.docx]

**Supplemental data 1**. Research terms

(("colorectal neoplasms"[MeSH Terms] OR ("colorectal"[All Fields] AND "neoplasms"[All Fields]) OR "colorectal neoplasms"[All Fields] OR ("colorectal"[All Fields] AND "cancer"[All Fields]) OR "colorectal cancer"[All Fields]) AND ("liver neoplasms"[MeSH Terms] OR ("liver"[All Fields] AND "neoplasms"[All Fields]) OR "liver neoplasms"[All Fields] OR ("liver"[All Fields] AND "cancer"[All Fields]) OR "liver cancer"[All Fields] OR ("liver"[All Fields] AND "metastasis"[All Fields]) OR "liver metastasis"[All Fields])) AND ("treatment"[MeSH Terms] OR "treatment"[All Fields] OR "therapeutics"[MeSH Terms] OR "therapeutics"[All Fields] OR "therapy"[All Fields]) AND (("systematic review"[Publication Type] OR "systematic reviews as topic"[MeSH Terms] OR "systematic"[All Fields] AND "review"[All Fields] OR "systematic review"[All Fields]) OR ("meta-analysis"[Publication Type] OR "meta-analysis as topic"[MeSH Terms] OR "meta-analysis"[All Fields])).
